# Pulmonary and intestinal microbiota dynamics during Gram-negative pneumonia-derived sepsis

**DOI:** 10.1186/s40635-021-00398-4

**Published:** 2021-07-12

**Authors:** Nora S. Wolff, Max C. Jacobs, W. Joost Wiersinga, Floor Hugenholtz

**Affiliations:** 1 Center for Experimental and Molecular Medicine, Amsterdam Infection & Immunity Institute, Amsterdam UMC, Location AMC, University of Amsterdam, Meibergdreef 9, 1105 AZ Amsterdam, The Netherlands; 2 Department of Medicine, Division of Infectious Diseases, Amsterdam UMC, Location AMC, University of Amsterdam, Meibergdreef 9, 1105 AZ Amsterdam, The Netherlands

**Keywords:** Pneumonia, Sepsis, *Klebsiella pneumoniae*, Microbiome, Lung microbiota, Gut microbiota, Tongue, Mice, Dynamics over time

## Abstract

**Background:**

The gut microbiome plays a protective role in the host defense against pneumonia. The composition of the lung microbiota has been shown to be predictive of clinical outcome in critically ill patients. However, the dynamics of the lung and gut microbiota composition over time during severe pneumonia remains ill defined. We used a mouse model of pneumonia-derived sepsis caused by *Klebsiella pneumoniae* in order to follow the pathogen burden as well as the composition of the lung, tongue and fecal microbiota from local infection towards systemic spread.

**Results:**

Already at 6 h post-inoculation with *K. pneumoniae*, marked changes in the lung microbiota were seen. The alpha diversity of the lung microbiota did not change throughout the infection, whereas the beta diversity did. A shift between the prominent lung microbiota members of *Streptococcus* and *Klebsiella* was seen from 12 h onwards and was most pronounced at 18 h post-inoculation (PI) which was also reflected in the release of pro-inflammatory cytokines indicating severe pulmonary inflammation. Around 18 h PI, *K. pneumoniae* bacteremia was observed together with a systemic inflammatory response. The composition of the tongue microbiota was not affected during infection, even at 18–30 h PI when *K. pneumoniae* had become the dominant bacterium in the lung. Moreover, we observed differences in the gut microbiota during pulmonary infection. The gut microbiota contributed to the lung microbiota at 12 h PI, however, this decreased at a later stage of the infection.

**Conclusions:**

At 18 h PI, *K. pneumoniae* was the dominant member in the lung microbiota. The lung microbiota profiles were significantly explained by the lung *K. pneumoniae* bacterial counts and *Klebsiella* and *Streptococcus* were correlating with the measured cytokine levels in the lung and/or blood. The oral microbiota in mice, however, was not influenced by the severity of murine pneumonia, whereas the gut microbiota was affected. This study is of significance for future studies investigating the role of the lung microbiota during pneumonia and sepsis.

**Supplementary Information:**

The online version contains supplementary material available at 10.1186/s40635-021-00398-4.

## Background

Lower respiratory tract infections account for more deaths than any other infectious disease [1]. *Klebsiella pneumoniae* is one of the most common Gram-negative bacterial causes of pneumonia and is increasingly difficult to treat due to the emergence of antibiotic resistance [2, 3]. Pneumonia-induced sepsis caused by *K. pneumoniae* is particularly known for its associated high rates of morbidity and mortality [4–6].

We and others have previously described the protective role of the gut microbiota during bacterial pneumonia caused by *Mycobacterium tuberculosis*, *Streptococcus pneumoniae*, *Staphylococcus aureus*, *Burkholderia pseudomallei* as well as *K. pneumoniae* [7–11]. It has been hypothesized that gut-derived metabolites, such as pathogen-associated molecular patterns (PAMPs) and short-chain fatty acids (SCFAs) aid in the pulmonary host defense by enhancing local antimicrobial functions in innate immune effector cells [8, 12–15]. Most recent evidence highlights the role of the commensal microbes of the respiratory tract in the development of pneumonia [16]. It was long believed that healthy lungs were a sterile environment, however, during the last decade research has shown that the lung also has its own microbiome [17, 18]. Recently, Dickson et al*.* [19] showed that the lung microbiome at admission was predictive of clinical outcome in ICU patients. Moreover, changes in the composition of the lung microbiome have been associated with susceptibility to influenza virus infection, the development of acute respiratory distress syndrome (ARDS) as well as poor outcome in patients with ARDS [20, 21].

In healthy lungs the main source of bacteria comes from the oropharynx microbiome. The main genera of bacteria found in the lower airways are *Veillonella*, *Prevotella*, and *Streptococcus*. Acute and chronic lung disease can severely change the lung microbiota. Often, the microbiota shifts away from the dominant Bacteroidetes phylum towards a bacteria from the Gammaproteobacteria, which contain many of the pneumonia-causing Gram-negative pathogens [18].

The main source of microbial organisms in healthy lungs is via microaspiration, primarily from the mouth [22]. During disease the main microbial source that determines the lung microbiome can change, for example; an overgrowth of an aspirated pathogen in an infection [23] or the enrichment of gut-derived species in critical ill patients [21]. Interestingly, pulmonary inflammation can also influence the composition of the gut microbiome. We have previously observed that severe pneumonia can results in significant shifts in the gut microbiota profile in a mouse model of melioidosis [24].

The changes in the composition of the lung microbiota during pneumonia are ill defined. To our knowledge, the only study that investigated respiratory microbiome dynamics over time focused on *Streptococcus pneumoniae* infection in young and elderly mice [25]. In that study, differences in microbiome composition between naive young-adult and elderly mice were associated with differences in pneumococcal clearance over time. However, this study focused on pathogen acquisition and clearance with a low bacterial inoculum, all mice recovered and cleared the pathogen from the lung 28 days after inoculation.

**Fig. 1:**
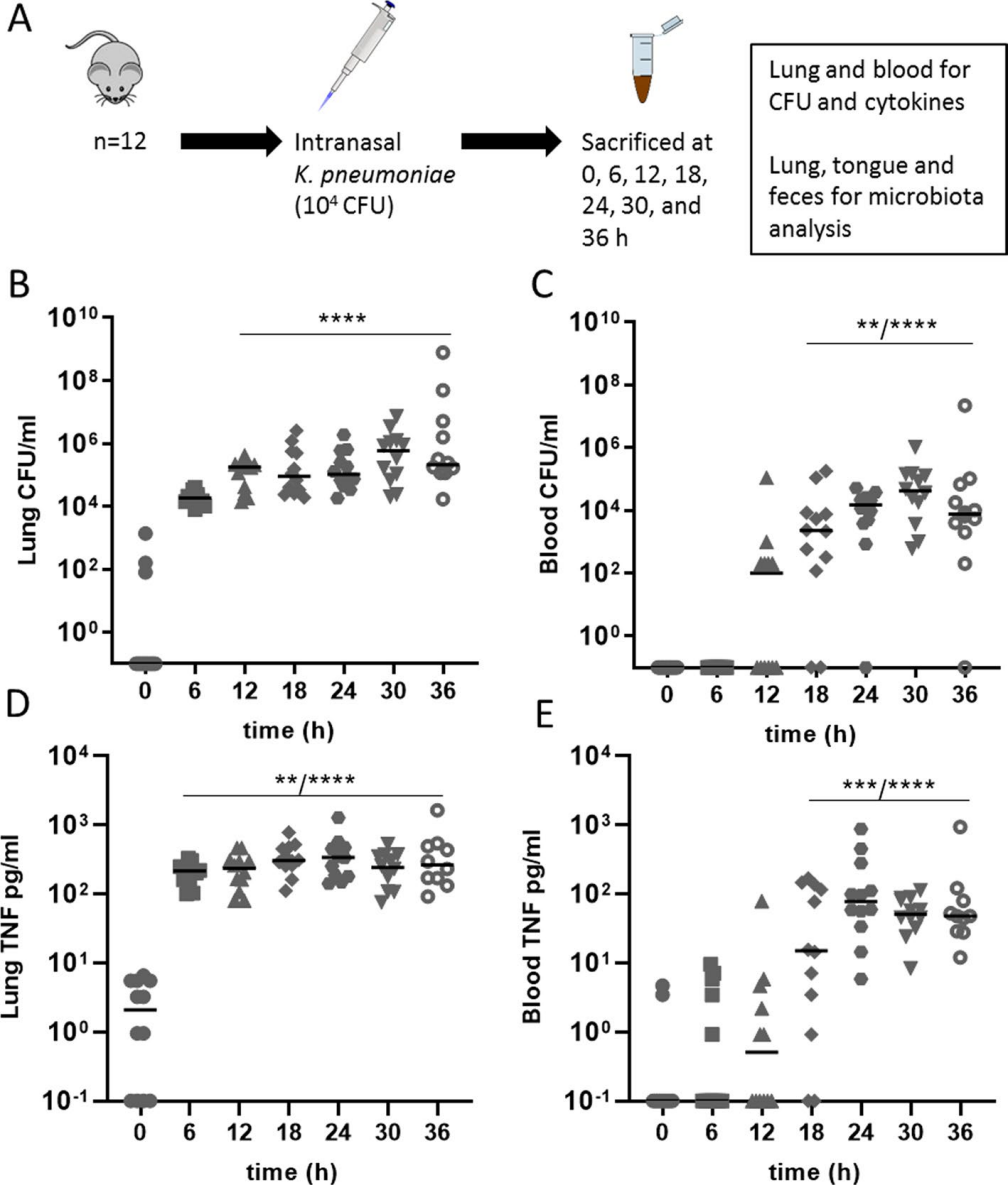
Organ bacterial loads and cytokine levels during *K. pneumoniae* infection. **a** Experimental design. Mice received an intranasal inoculation with 10^4^ colony forming units (CFU) of *K. pneumoniae*. A group was killed every 6 h, until 36 h (*n* = 10–12) at which time blood, lung, tongue and feces were collected, *t* = 0 serves as the control group. This model becomes lethal approximately 40 h post-inoculation (**a**). Pulmonary (**b**) and blood (**c**) CFU at 0 to 36 h post-inoculation. Tumor necrosis factor (TNF) of lung homogenate (**d**) and blood plasma (**e**) at 0 to 36 h post-inoculation. Data are shown as median, the top bar denotes at which time-points the data are significantly different from the 0-h group, the stars show the range of significance, *p* < 0.01 (**), *p* < 0.001 (***) and *p* < 0.0001 (****)

We hypothesized that the murine lung microbiota changes during experimental *K. pneumoniae* pneumonia. In addition, we investigated whether the tongue can be used as a proxy to lung microbiota and whether the tongue and fecal microbiota change and influence the lung microbiota during infection. Mice were inoculated with *K. pneumoniae* and killed every 6 h up to 36 h post-inoculation to determine both local and systemic bacterial counts and inflammation in addition to the lung-, tongue- and gut microbiome dynamics over time (Fig. 1a). This study will aid in understanding the lung microbiota dynamics at the onset and during pneumonia as well as its relation with the gut and oral microbiota.

## Methods

### Mice

Specific pathogen-free C57BL/6J male and female mice were ordered Charles River (C57BL/6J, Sulzfeld, Germany) and housed in groups in individually ventilated cages enriched with disposable rodent homes and nestling paper. All mice received the Teklad 2916 diet ad libitum for the full length of the experiment. All mice were housed at the Animal Research Institute AMC (ARIA) of our institution. They acclimatized on site for 2 weeks prior to the start of the experiments, at which time the mice were between 10 and 11 weeks of age and in good health. Mice were assessed on their welfare (incl. posture and activity) throughout their stay at the facility by both the researchers and the animal care takers.

### Study design

Each experimental group consisted of 12 mice (6 males, 6 females, 84 mice total) spread across 4 cages, genders were housed separately. The number of animals was determined through sample size calculations using previous data from murine pneumonia experiments, focusing on colony forming units (CFU). The calculations were performed for 80% power and effect size 1.85, with a significance level of 0.05 [26–28]. Pulmonary infection was induced by intranasal inoculation of 10^4^ CFU of *K. pneumoniae* serotype 2 (ATCC 43816), dissolved in 50 µl phosphate buffered saline as described [26, 27, 29]. The control group (*t* = 0) received only the 50 µl phosphate buffered saline. This well-established *K. pneumoniae* infection model leads to a marked lung infection 12 h post-inoculation. Hereafter, the infection disseminates to other body sites and becomes lethal after approximately 2 days [26, 28, 30, 31]. Inoculation was performed under a mild sedation with 2–3% isoflurane in 100% O_2_, to ensure that the mice would calmly breathe in the fluids. Due to logistic reasons, the mice were inoculated in two different batches: one batch consisted of *t* = 0, *t* = 12, *t* = 18 and *t* = 36 h, the second batch consisted of the time-points *t* = 6, *t* = 24 and *T* = 30 h. Mice were euthanized at 6, 12, 18, 24, 30 and 36 h post-inoculation as described [26, 29], and the control group simultaneously with the last time-point. Euthanasia was performed using an intraperitoneal injection of ketamine and dexmedetomidine (a combination was made with 12.5 mg/ml ketamine, 30 µg/ml dexmedetomidine of which the mice were given 0.1 ml/10 g mice) followed by cardiac puncture. During the experiments no mice reached the predetermined humane endpoint (based on ARIA’s general scoring system including breathing, activity and posture), and no mice died during infection. The experiments were designed following the suggested guidelines of Osuchowski et al*.* [32].

### Sample collection, processing and assays

Of all mice, fresh feces were collected just before inoculation, snap-frozen in liquid nitrogen and stored at − 80 °C. Blood from the cardiac puncture was collected in heparin and immediately cooled. A lung and tongue were snap-frozen in liquid nitrogen and stored at − 80 °C. Furthermore, the other lung was homogenized in isotonic saline (4 ml per gram of tissue). Bacterial loads of the lung and blood were determined by serial dilution plated onto sheep-blood agar plates and incubated for 16 h at 37 °C, after which the CFU were counted. Following the plating, the blood was centrifuged at 3000 rpm for 10 min at 4 °C, in order to obtain the blood plasma. The lung homogenates were diluted 1:1 with a lysis buffer (1% (v/v) Triton X-100, 150 mM NaCl, 15 mM Tris, 1 mM MgCl(H_2_O)_6_, 1 mM CaCl_2_(H_2_O)_2_, pH 7.4) including a protease inhibitor (complete protease inhibitor cocktail tablets, Roche, Basel, Switzerland) and incubated on ice for 30 min, followed by centrifugation at 4000 rpm, for 10 min at 4 °C, after which the supernatant was stored. In blood plasma and lung homogenate supernatant, interleukin (IL)-6, tumor necrosis factor (TNF)-α, monocyte chemoattractant protein (MCP)-1 and interferon (IFN)-γ levels were determined by a cytometric bead array multiplex (the Mouse Inflammation Kit, BD Biosciences, New Jersey, USA).

### Microbiota profiling

Repeated bead-beating of the lung, tongue and fecal pellets was performed as described elsewhere (protocol 5 of Costea et al. [33], with STAR (Stool transport and recovery) buffer (Roche, Basel Switzerland)). Following centrifugation, 250 µl supernatant was used with the Maxwell® RSC Blood DNA Kit (Promega, Madison, USA), and the DNA was eluted in 50 µl DNAse free water. Twenty nanograms of DNA was used for the amplification of the V3–V4 region of the 16S rRNA gene as described [34], with barcoded 341 forward and 805 reverse primers for 25 cycles. For the purification of the amplified product, the AMPure XP beads (Beckman Coulter, Indianapolis, USA) were used according to manufacturer’s guidelines on a Beckman Coulter Biomex FX. The purified product was equimolar mixed and loaded for sequencing on the Illumina MiSeq with the MiSeq V3 – 600 cycle kit, as instructed by Illumina. The sequence reads were analyzed as follows. Read pairs with perfect matching forward and reverse barcodes were assigned to their corresponding samples. The forwards and reverse reads were length trimmed at 240 and 210, respectively, which were inferred and merged with amplicon sequence variants (ASVs) using DADA2 V.1.5.2 [35]. The assignment of taxonomy was done using the DADA2 implementation of the RDP classifier [36] and SILVA 16S reference database [37]. Before the statistical testing (see “Statistical analysis” section below) the data were first screened for an in-house list of common lab-contaminants, if detected these sequences were removed from the dataset.

### Statistical analysis

Microbiome statistical tests were performed using the vegan, phyloseq and microbiome package in R. For alpha diversity calculations (Observed species, Chao1 index and Shannon index) the microbiota was rarefied at 500 sequences for lung samples and at 30,000 sequences for tongue and fecal samples. Principal coordinates analysis (PCoA) of Bray–Curtis dissimilarities was used to calculate beta diversity using non-rarefied data. The composition plots, principal response curve (PRC), Permanova and Spearman correlations were done on genus-level relative abundance data. The PRC places time on the *x*-axis and takes time along as a co-variate in the analysis and takes another variable, here the infection, to create a new variable which is the interaction of infection and time (creating infection × time). The average genera abundances were calculated between the mice from different time-points during the infection and compared per time-point to *t* = 0, where the average of *t* = 0 was copied at each time-point to create an arbitrary group which could be placed on zero on the *x*-axis. To assess the significance of each of the PRC axes hierarchical permutation tests were performed and *p*-values calculated. To determine the origin/source (where the bacteria come from) of the lung microbiota, we used FEAST (fast expectation–maximization microbial source tracking). This uses ASV count data of the fecal, tongue and lung microbiome. The individual sample source percentages were calculated using the FEAST probabilistic model and an expectation–maximization approach as described by Shenhav et al. [38]. Statistical analysis on the bacterial counts, inflammatory markers and FEAST generated data was performed using GraphPad Prism 8 software. Significance was calculated using the Kruskal–Wallis one-way ANOVA with an uncorrected Dunn’s test and verified by a false discovery rate analysis of Benjamini and Hochberg. *p*-values < 0.05 were considered statistically significant.

## Results

### Dynamics of local and systemic bacterial loads and inflammation during *K. pneumoniae* pneumonia

To investigate the changes of the lung microbiota during infection, we used our well-established mouse model of *Klebsiella pneumoniae*-induced pneumonia [26, 29]. The lung, tongue and gut were sampled at multiple time-points during infection to determine both local and systemic bacterial counts and inflammation in addition to the long, tongue and gut microbiome dynamics over time (Fig. 1a).

A rapid logarithmic increase in lung bacterial counts of *K. pneumoniae* was seen from 6 h post-inoculation (PI) onwards, plateauing after approximately 18 h (Fig. 1b)*.* The first blood cultures became positive 12 h PI, reaching significant differences from *t* = 0 at 18 h PI (Fig. 1c). This was also reflected in pro-inflammatory cytokine profiles with a strong increase of TNF-α, IFN-γ, IL-6 and MCP-1 levels appearing first locally in the lung after which all mice demonstrated elevated levels of these cytokines in blood already 18 h PI, which emphasizes the systemic inflammatory response syndrome seen in this model (Fig. 1d, e, Additional file 1: Fig. 1).

### Impact of the *K. pneumoniae* infection on the lung microbiota

**Fig. 2:**
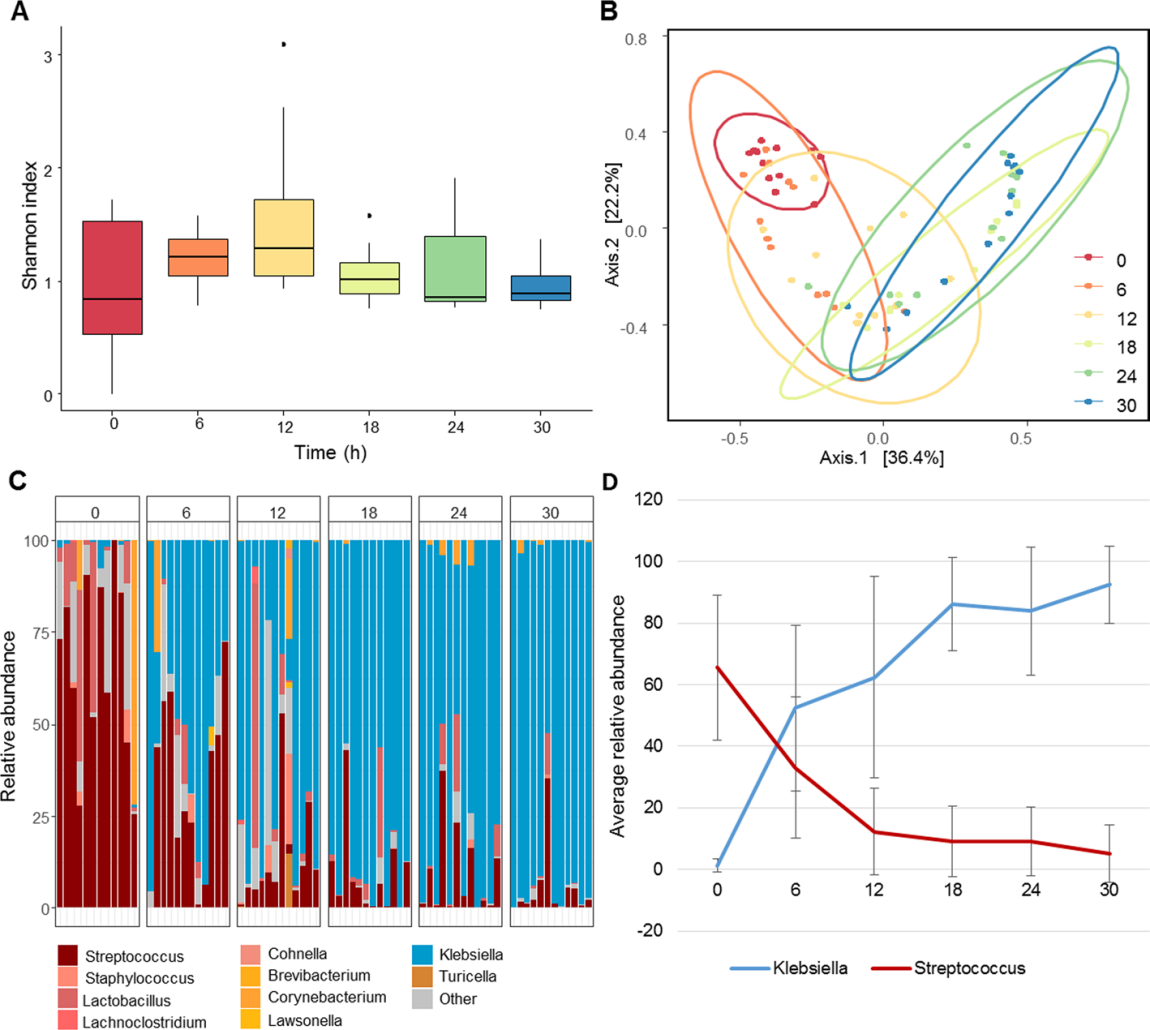
Lung microbiota dynamics after inoculation with 1 × 10^4^ CFU of *K. pneumoniae*. Samples were analyzed at time is 0, 6, 12, 18, 24 and 30 h, with *n* = 12 mice. **a** Shannon index for alpha diversity. **b** Principal coordinates analysis (PCoA) of Bray–Curtis dissimilarities of the lung microbiota. **c** Relative abundances of top 10 most abundant genera. **d** The most prevalent genera *Streptococcus* and *Klebsiella* have been plotted with their relative abundances and standard deviations over time

In order to evaluate the impact of *K. pneumoniae* infection on the lung microbiome, we first sequenced the lung at all time-points. Alpha diversity of the lung microbiota was not affected by the infection compared to baseline microbiota diversity at *t* = 0 as measured by Chao1, observed species and Shannon's index (Fig. 2a, Additional file 2: Fig. 2A). However, beta diversity showed that the microbial composition of the lung shifted away from healthy control mice during infection (Fig. 2b). In the healthy lung microbiota (*t* = 0 h PI) *Streptococcus* was the most abundant genus present at 65.6% relative abundance, and *Lactobacillus* was present at 11.6% relative abundance. The change in the lung microbiota over time was largely driven by increased *K. pneumoniae* bacterial counts in almost all the mice from 18 h PI onwards (Fig. 2c, Additional file 2: Fig. 2B). At 12 h PI other genera also appeared in larger numbers, such as taxa from the *Lachnospiraceae* and *Staphylococcus.* To confirm our observation in microbial shift both in the composition plot and the overall shift observed in the beta diversity, we performed a principal response curve (PRC). The PRC focusses on the effect the infection has on the microbiota composition over time, and it calculates which bacterial genera are important in explaining the differences, where the microbiota composition of the baseline (*t* = 0) mice were used as the non-infected group (Additional file 2: Fig. 2C). This clearly showed that the genus *Streptococcus* and *Klebsiella* were the most prominent members over time, also illustrated in Fig. 2d where we plotted the average relative abundances of these species over time.

### Correlation of pulmonary microbiota to lung bacterial counts and cytokine levels during infection

Furthermore, to investigate whether the composition of the lung microbiota was correlated to time post-inoculation, bacterial counts (as measured in CFUs) and pulmonary cytokine levels, we performed a permutational multivariate analysis of variance (Permanova). Using Bray–Curtis dissimilarities of the microbiota between samples, the Permanova fits linear models to the variables and calculates with permutation testing weather the variable is significantly correlated to the microbiota (Table 1). The significant variables were time-points, CFUs of the lung and TNF-α levels of lung homogenates, of which we calculated the top taxa involved in the correlation (Table 1 and Additional file 3: Fig. 3A–C). These analyses confirmed *Klebsiella* and *Streptococcus* as major players. Moreover, to investigate if single members of the microbiota were correlated to these same variables, Spearman correlations were performed (Additional file 3: Fig. 3D). Among the significantly correlating genera were *Klebsiella* and *Streptococcus*, but also *Pseudomonas*. However, as also shown in Fig. 2c *Pseudomonas* was a minor species in the lung microbiota, not represented in the top 10 most abundant genera in the lung.

**Table 1:** Time post-inoculation, pulmonary bacterial counts and TNF-α correlate to the lung microbiota over time

	*p*-value	*F* model	*R*^2^
Murine gender	0.689	0.420	0.006
CFU in blood	0.876	0.765	0.399
MCP-1	0.618	0.830	0.966
IFN-y	0.220	1.183	0.317
IL-6	0.191	1.590	0.936
TNF-α	0.044	1.877	0.875
CFU in lung	0.008	2.263	0.852
Time post-inoculation	0.001	15.905	0.546

Values were calculated using Permanova and used ASV level data for the lung microbiota, the cytokines were measured in lung homogenates

*ASV* amplicon sequence variance, *CFU* colony forming unit, *MCP-1* monocyte chemoattractant protein 1, *IFN-γ* interferon γ, *IL-6* Interleukin 6 and *TNF-α* tumor necrosis factor α

### Dynamics of the tongue and gut microbiome during *K. pneumoniae*-induced pneumosepsis

**Fig. 3:**
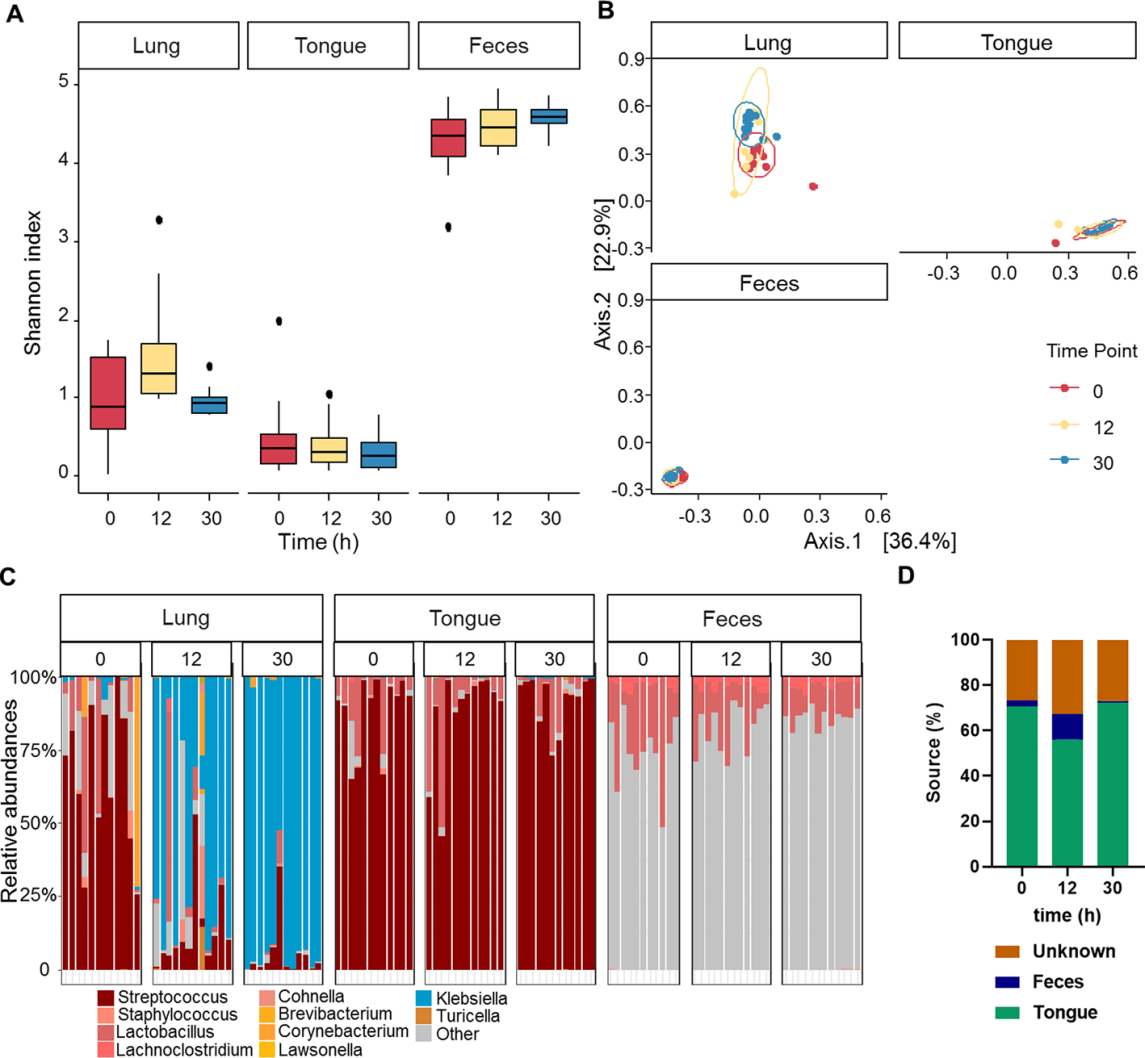
Alpha and beta diversity of the lung, tongue and fecal microbiotas. Mice received an intranasal inoculation with 10^4^ colony forming units (CFU) of *K. pneumoniae*. Mice were killed at 0, 12, and 30 h post-inoculation (n = 12), lung tongue and feces were extracted for microbiota analysis. **a** Alpha diversity; Shannon of lung tongue and fecal samples at 0, 12 and 30 h post-inoculation. **b** Beta diversity; PCoA Bray analysis of lung tongue and feces over time. **c** Genus distribution for lung tongue and fecal microbiota at 0, 12, 30 h post-inoculation, where the top 10 genera in the lung are colored, other genera are grey. **d** Fast expectation–maximization for microbial source tracking (FEAST) analysis on the lung microbiota showing which percentage of the lung microbiota can be traced back to the tongue, feces and unknown source at 0, 12 and 30 h post-inoculation, in a dataset without *K. pneumoniae*

To investigate the tongue and fecal microbiota during *K. pneumoniae* pneumonia, we sampled the tongue (as an indicator of oral microbiota) and feces of the mice at 0, 12 and 30 h after intranasal inoculation. In contrast to the lung microbiome, on which the *K. pneumoniae* infection had a profound impact, we did not observe major changes in the alpha and beta diversity and the relative abundances of phylum and genus over time in the tongue and fecal microbiota (Fig. 3a–c, Additional file 4: Fig. 4A–C). However, Permanova analysis on the relative abundances at the genus and ASV level showed a significant difference between the time-points (*p* = 0.02 and *p* = 0.01, respectively) for the fecal samples. The tongue samples were not significantly different between the time-points (*p* = 0.53, *p* = 0.55 at genus and ASV level, respectively).

To investigate the origin of the lung microbiota, we used the fast expectation–maximization for microbial source tracking (FEAST) analysis. This analysis was performed without *K. pneumoniae* present in the dataset, as the source of that is external, since it was administrated by us. Moreover, *K. pneumoniae* was hardly present in the tongue and gut (Fig. 2c) at 12 or 30 h PI. This analysis showed that the healthy lung microbiota largely resembles that of the tongue, but not fecal microbiota. Minimal changes were seen in the contribution of the tongue microbiota to the lung, whereas the fecal microbiota showed a higher contribution to the lung microbiota at 12 h PI, in comparison to 30 h PI (*p* < 0.001, Fig. 3d, Additional file 4: Fig. 4D and E).

## Discussion

We aimed to demonstrate the dynamics in microbiotas during murine *K. pneumoniae* pneumonia. Profound changes in the lung microbiota were seen already 6 h post-inoculation. Alpha diversity of the lung microbiota was not affected by the infection, whereas the beta diversity was. During infection we saw a constant multiplication of *Klebsiella*, whereas the amount of *Streptococcus* present in the lung decreased. Furthermore, in order to investigate the source of the lung microbiota during infection, we also sequenced tongue and fecal samples. Unlike the tongue microbiota, we did see a contribution of the fecal microbiota to the lung microbiota early on in the infection, but this decreased at a later stage in the infection (30 h post-intranasal inoculation).

The lung microbiome has previously been described to closely resemble that of the oropharynx, and contains species such as *Streptococcus*, *Veillonella* and *Prevotella,* which take up 20–30% of the microbiota composition [17, 22]. In our study, we observed that in the healthy murine lung *Streptococcus* was the most abundant genus present (65.6% relative abundance), while *Lactobacillus* was also present in all mice (11.6% relative abundance). This is in contrast to Krone and colleagues [25] who analyzed murine lungs during a *Streptococcus pneumoniae* infection. They found a large contribution of several *Bacteroidetes* genera in the healthy murine lungs, but also during and after clearance of a *Streptococcus pneumoniae* infection. They did not observe large contributions of *Streptococcus* or *Lactobacillus* in the healthy murine lung. These differences in the baseline composition of the microbiome could be explained by vendor differences by breeders, or possible differences between batches of mice [29, 32, 39]. However, these differences at baseline do need to be addressed when translated to human diseases, such as *Klebsiella pneumoniae* infections in humans.

In a healthy lung the microbiota is delicately balanced by the reproduction rate of present bacteria, and the immigration and elimination of bacteria. Under normal circumstances, the reproduction rate of bacteria remains low and the immigration and elimination high. However, in (chronic) airway diseases, such as chronic obstructive pulmonary disease, asthma, and cystic fibrosis, a shift occurs in this balance leading to changes in the microbiota [40, 41]. This can decrease microbial elimination and provide a nutrient-rich environment and areas of lower oxygen levels, allowing bacteria to thrive in the lung. Furthermore, many of the therapies that are often administered during hospitalization for pneumonia can affect the composition of the microbiome, including the use of antibiotics and corticosteroids [17, 18, 41–43]. In critical illness the composition of the lung microbiota shifts from closely resembling the oropharynx microbiota towards resembling that of stomach and small intestines, therefore changing the main source of microbes for the lung microbiota [18, 21, 42]. It is important to know the source of bacteria for the lung microbiota as there is a growing body of evidence that the lung microbiota changes during disease and can even predict the outcomes of some diseases [19–21, 40]. Therefore, knowing where the unwanted microbes come from can potentially lead to a different view of current treatments.

Fast expectation–maximization for microbial source tracking (FEAST) analysis was used to investigate whether there was a shift in origin of the microbiota of the lung towards that of the tongue or feces overtime. The tongue microbiota showed large similarities with the lung microbiota in a healthy state. However, we were surprised by how little the tongue microbiota changed over the course of the infection and remained the main source for the lung microbiota. Whereas, the gut microbiota started to contribute to the lung microbiota at 12 h post-inoculation, however, this decreased at 30 h post-inoculation when the infection was severe. This murine model of pneumonia and severe sepsis did not show that the main source of the lung microbiota changed towards that of the fecal microbiota during critical illness. Future experiment of the small intestine and stomach during a murine *K. pneumoniae* infection could be interesting to see whether that would replicate the earlier described transition of source for the lung microbiota in the critically ill [18, 21, 42]. It is also important to note that this study was performed using a murine model in which we did not replicate treatments that human patients would get in hospital, such as sedatives and opiates which potentially affects physical activity and gastrointestinal motility [42].

This study has a number of limitations. First of all, 16S rRNA sequencing was used to analyze the microbiotas, which gives an idea of which bacteria were present in these tissues, but does not give us any functional information. Other methods such as metagenomics and metatranscriptomics, would have also given us additional functional information. Using 16S rRNA sequence analysis only provides data on the bacterial composition, but we did not analyze the composition of fungi and viruses and whether they change in the lung during *K. pneumoniae* pneumonia. Furthermore, this study investigated the fecal microbiota using fresh stool, whereas it would be interesting to study the upper intestinal tract to gain a better insight into its role as a source for the lung microbiome during critical illness. Moreover, in murine studies, as mentioned above, vendor, diet and batch effect can give rise to differences in these microbiotas [29, 32, 39]. The results are based on a Gram-negative pneumonia that was induced by one strain of *K. pneumoniae*, whereas different clinical isolates of *K. pneumoniae* can lead to variations in innate immune response [44], potentially affecting the microbiotas. These experiments were set up using mice, which has as a benefit that it allows us to examine whole organs at many specific time-points post-inoculation which would not be possible in humans. Nonetheless, it is limited in translational capabilities to humans as the composition of the microbiota can see similar trends, but remains different [39].

## Conclusions

In conclusion, this study served to gain greater insight into the murine lung microbiota during Gram-negative *K. pneumoniae* infection, showing the take-over of the infectious pathogen and the lowering abundance of *Streptococcus*. Furthermore, unlike in humans, this study did not see that the oral microbiota is a good proxy for the lung microbiota. We did demonstrate changes of the gut microbiota during pneumonia, which might be a good predictor for the severity of pneumonia in less lethal pneumonia models. This paper is of significance for future studies investigating the role of the lung microbiome during pneumonia and sepsis.

## Supplementary Information

**Additional file 1: Fig. 1.** Cytokine values of lung and blood during *K. pneumoniae *infection. Mice received an intranasal inoculation with 10^4^colony forming units (CFU) of *K.pneumoniae*. A group was sacrificed every 6 hours, until 36 hours (n=10-12).Interferon (IFN)-γ (**A**, **B**), interleukin (IL)-6 (**C**, **D**),and monocyte chemoattractant protein -1 (MCP-1) (**E**, **F**) were measuredin lung homogenate (**A**, **C** and **D**) and blood plasma (**B**, **D**and **F**). Data is shown as median, thetop bar denotes at which timepoints the data is significantly different fromthe 0 hour group, the stars show the range of significance, P<0.05 (*),p<0.01 (**), p<0.001 (***), p<0.0001 (****).

**Additional file 2: Fig. 2.** Lung microbiota dynamics after inoculation with 1x10^4^ CFU of K. pneumoniae. Samples were analyzed at time is 0, 6, 12, 18, 24 and 30 hours, with n=12 mice. (A) Observed species, Chao1 index and Shannon index for alpha diversity. (B) Relative abundances of the phyla in the lung microbiota. (C) PRC places time on the x-axis and takes time along as a co-variate in the genera of the lung microbiota composition on T=0 was copied at each time point to create an arbitrary group which could be placed on zero on the x-axis. The lung microbiome was significantly different (p=0.01) in time than our arbitrary T=0 group om the x axis.

**Additional file 3: Fig. 3.** Top correlations to the lung microbiota overtime found by Permanova. The timespan of the experiment, the measured CFU’s, cytokines in the lung homogenate were tested for correlation with the lung microbiota by Permutational Multivariate Analysis of Variance (Permanova). Using bray-curtis dissimilarities of the microbiota between samples, the Permanova fits linear models to the variables and calculates with permutation testing weather the variable is significantly correlated to the microbiota (Table 1). The top taxa involved in the correlation with the significant variables are shown in the this figure for (A) time-points, (B) CFUs of the lung and (C) TNF-alpha levels. (D) Correlation to single members of the microbiota was tested via Spearman correlation to these same variables, + sign in the boxes indicate adjusted p-value > .1, increased intensity of red is a greater positive correlation and blue indicated a negative correlation. 

**Additional file 4: Fig. 4.** Relative abundance and beta diversity of lung tongue and fecal microbiota. Mice received an intranasal inoculation with 104 colony forming units (CFU) of K. pneumoniae. Mice were sacrificed at 0, 12, and 30 hours post inoculation (n=12), lung, tongue and feces were extracted for microbiota analysis. (A) Phylum distribution for lung tongue and fecal microbiota. PCoA of Bray-Curtis dissimilarities of tongue (B) and lung (C) microbiota over time. Fast expectation-maximization for microbial source tracking (FEAST) analysis on the lung microbiota showing which percentage of the lung microbiota can be traced back to the tongue (D) and feces (E) source in a data set without *K. pneumoniae*. Data is shown as median, p< 0.001 (***), n.s. denotes not significant.

## Data Availability

The datasets used and/or analyzed during the current study are available from the corresponding author on reasonable request.

## Abbreviations

ARDS: Acute respiratory distress syndrome
ASV: Amplicon sequence variant
CFU: Colony forming units
FEAST: Fast expectation–maximization microbial source tracking
ICU: Intensive Care Unit
IFN-γ: Interferon γ
IL-6: Interleukin 6
*K. pneumoniae*: *Klebsiella pneumoniae*
MCP-1: Monocyte chemoattractant protein 1
PCoA: Principal coordinates analysis
PRC: Principal response curve
Permanova: Permutational multivariate analysis of variance
PI: Post-inoculation
STAR: Stool transport and recovery
TNF-α: Tumor necrosis factor-alpha
