# Supplementary figures and images for: Pulmonary and intestinal microbiota dynamics during Gram-negative pneumonia-derived sepsis
Source: Intensive Care Med Exp. 2021 Jul 12;9:35. doi: 10.1186/s40635-021-00398-4 (PMC8272965; doi:10.1186/s40635-021-00398-4)

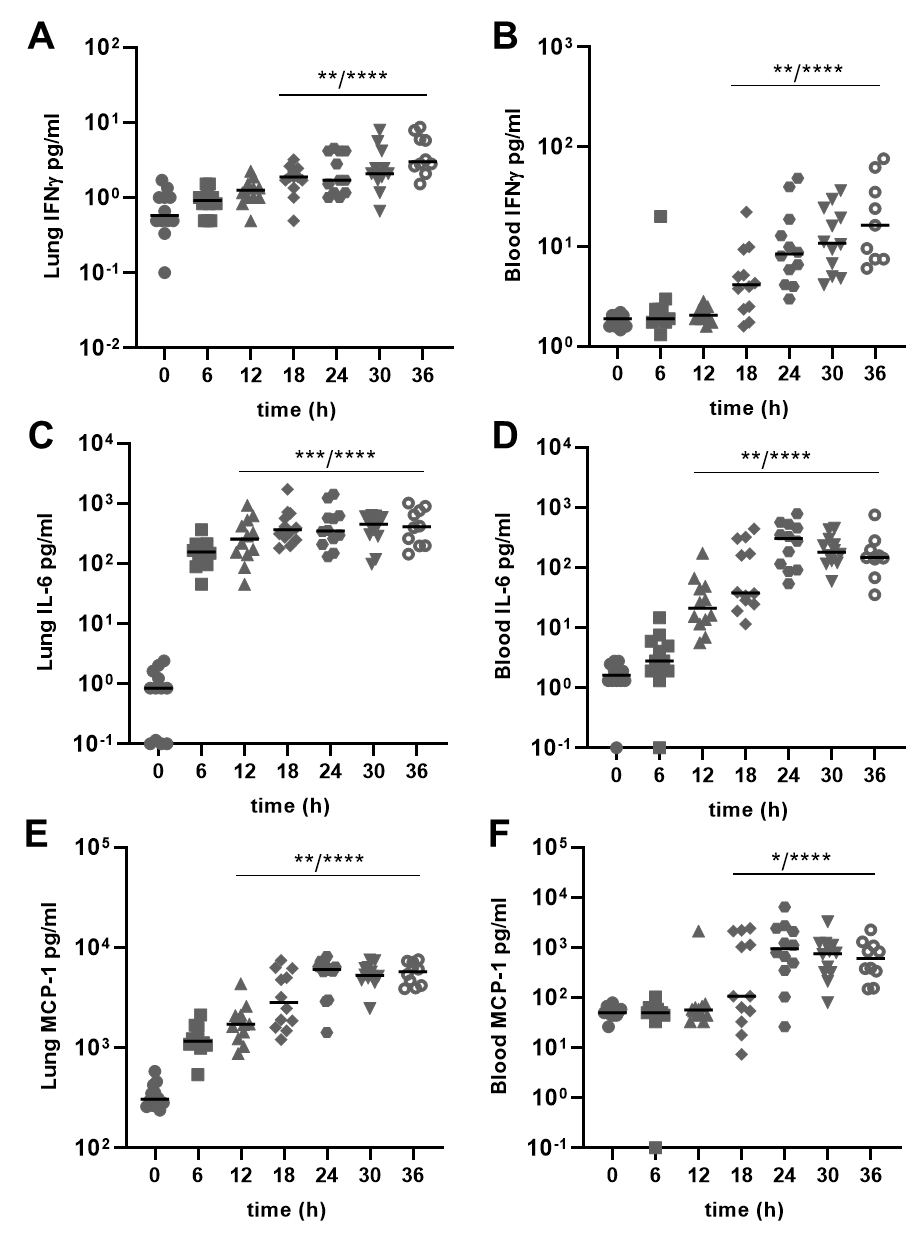

Supplement: Supplementary file 1 — Additional file 1: Fig. 1. Cytokine values of lung and blood during K. pneumoniae infection. Mice received an intranasal inoculation with 104colony forming units (CFU) of K.pneumoniae. A group was sacrificed every 6 hours, until 36 hours (n=10-12).Interferon (IFN)-γ (A, B), interleukin (IL)-6 (C, D),and monocyte chemoattractant protein -1 (MCP-1) (E, F) were measuredin lung homogenate (A, C and D) and blood plasma (B, Dand F). Data is shown as median, thetop bar denotes at which timepoints the data is significantly different fromthe 0 hour group, the stars show the range of significance, P<0.05 (*),p<0.01 (**), p<0.001 (***), p<0.0001 (****). [file 40635_2021_398_MOESM1_ESM.png]

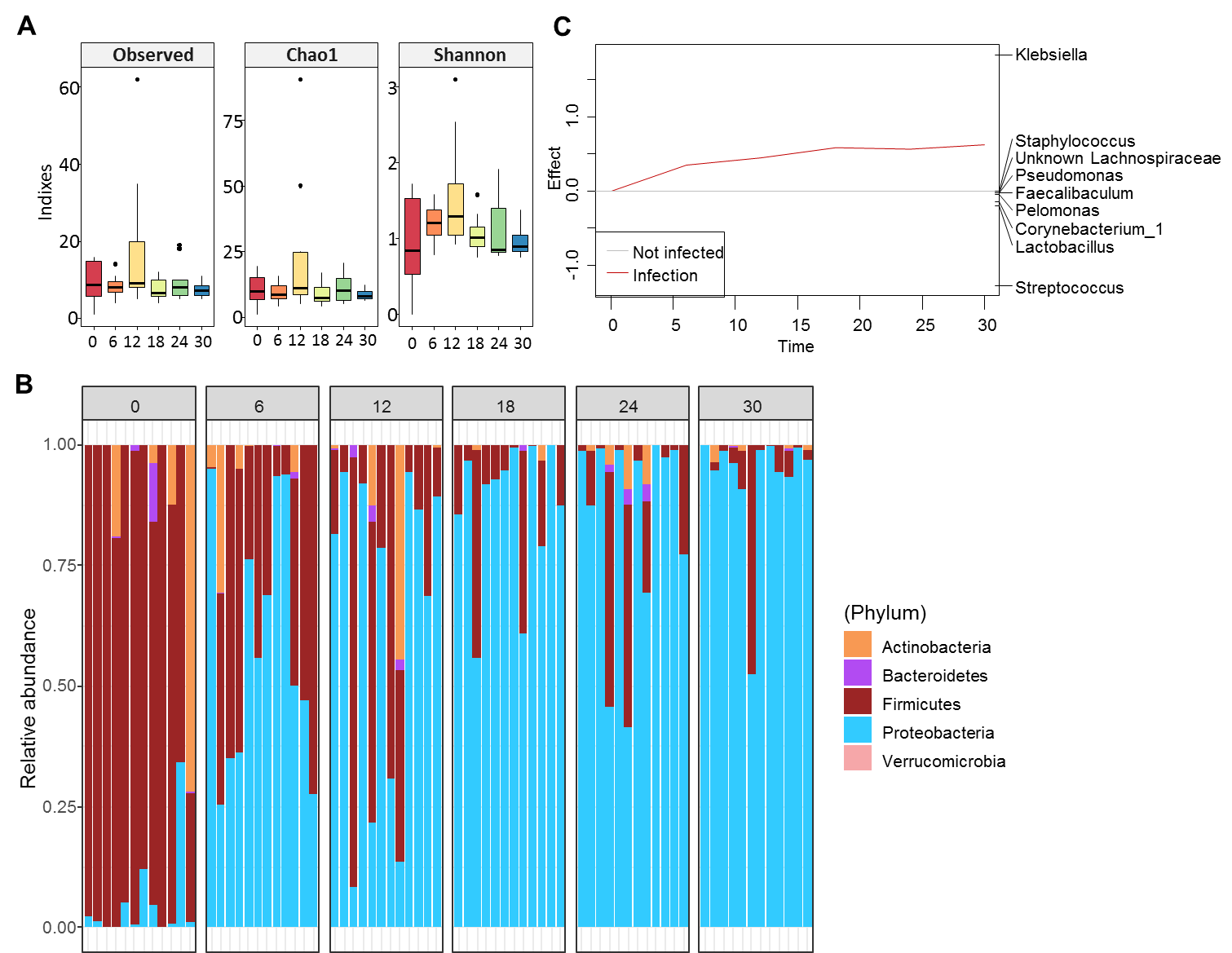

Supplement: Supplementary file 2 — Additional file 2: Fig. 2. Lung microbiota dynamics after inoculation with 1x10^4^ CFU of K. pneumoniae. Samples were analyzed at time is 0, 6, 12, 18, 24 and 30 hours, with n=12 mice. (A) Observed species, Chao1 index and Shannon index for alpha diversity. (B) Relative abundances of the phyla in the lung microbiota. (C) PRC places time on the x-axis and takes time along as a co-variate in the genera of the lung microbiota composition on T=0 was copied at each time point to create an arbitrary group which could be placed on zero on the x-axis. The lung microbiome was significantly different (p=0.01) in time than our arbitrary T=0 group om the x axis. [file 40635_2021_398_MOESM2_ESM.png]

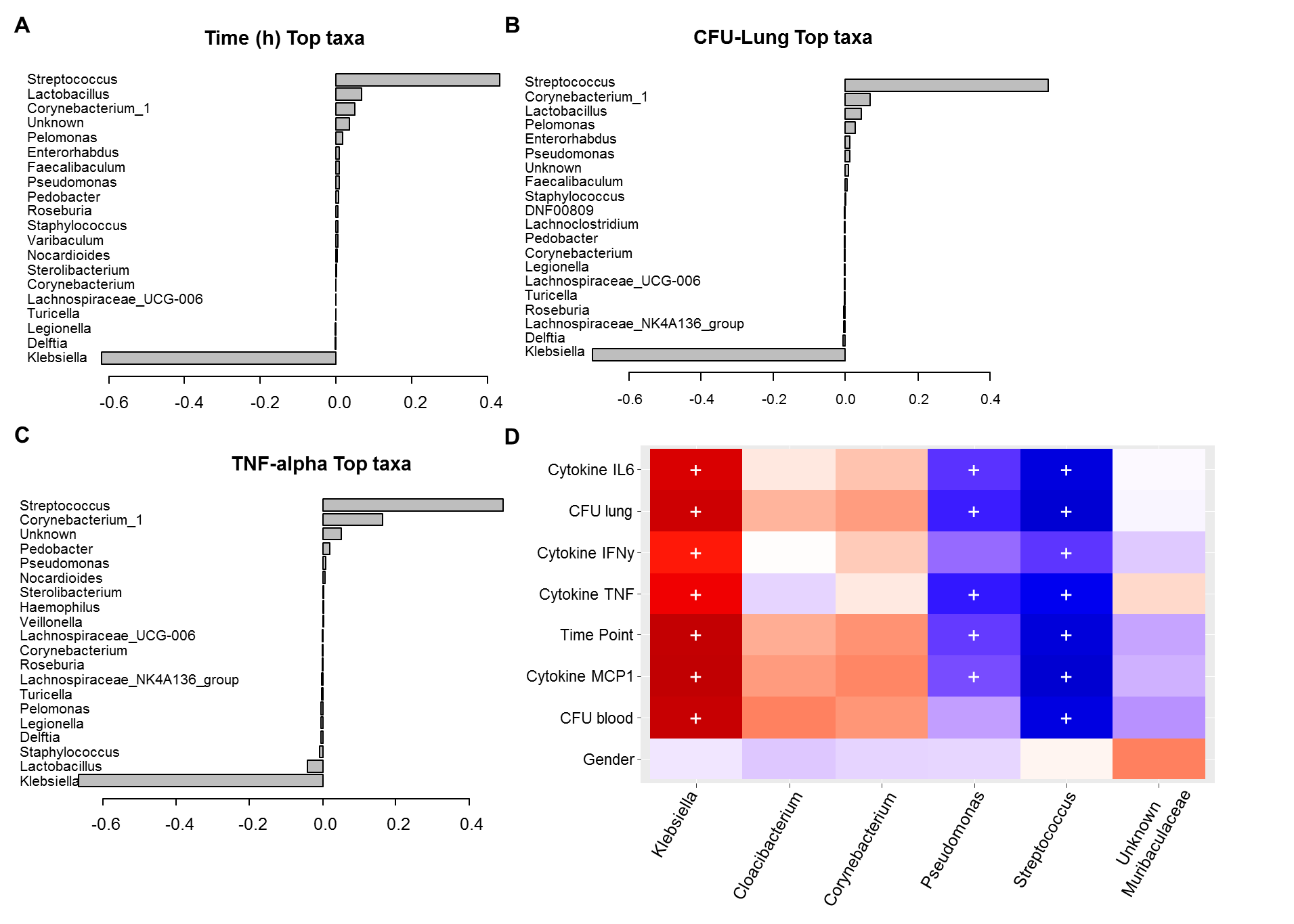

Supplement: Supplementary file 3 — Additional file 3: Fig. 3. Top correlations to the lung microbiota overtime found by Permanova. The timespan of the experiment, the measured CFU’s, cytokines in the lung homogenate were tested for correlation with the lung microbiota by Permutational Multivariate Analysis of Variance (Permanova). Using bray-curtis dissimilarities of the microbiota between samples, the Permanova fits linear models to the variables and calculates with permutation testing weather the variable is significantly correlated to the microbiota (Table 1). The top taxa involved in the correlation with the significant variables are shown in the this figure for (A) time-points, (B) CFUs of the lung and (C) TNF-alpha levels. (D) Correlation to single members of the microbiota was tested via Spearman correlation to these same variables, + sign in the boxes indicate adjusted p-value > .1, increased intensity of red is a greater positive correlation and blue indicated a negative correlation. [file 40635_2021_398_MOESM3_ESM.png]

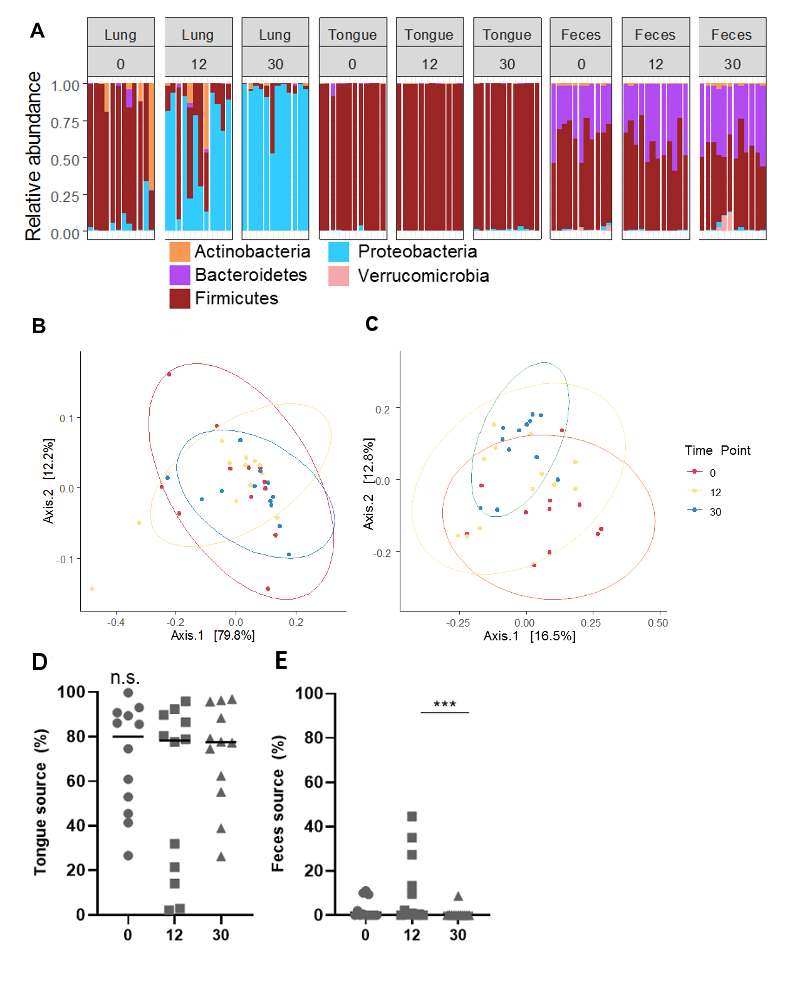

Supplement: Supplementary file 4 — Additional file 4: Fig. 4. Relative abundance and beta diversity of lung tongue and fecal microbiota. Mice received an intranasal inoculation with 104 colony forming units (CFU) of K. pneumoniae. Mice were sacrificed at 0, 12, and 30 hours post inoculation (n=12), lung, tongue and feces were extracted for microbiota analysis. (A) Phylum distribution for lung tongue and fecal microbiota. PCoA of Bray-Curtis dissimilarities of tongue (B) and lung (C) microbiota over time. Fast expectation-maximization for microbial source tracking (FEAST) analysis on the lung microbiota showing which percentage of the lung microbiota can be traced back to the tongue (D) and feces (E) source in a data set without K. pneumoniae. Data is shown as median, p< 0.001 (***), n.s. denotes not significant. [file 40635_2021_398_MOESM4_ESM.png]
